# Supplementary material for: Proposed Protocol for Field Testing of Endurance Fitness of Young Labrador Retrievers
Source: Methods Protoc. 2023 Jun 28;6(4):61. doi: 10.3390/mps6040061 (PMC10366876; doi:10.3390/mps6040061)
Supplement: Supplementary file 1 [file mps-06-00061-s001.zip › mps-2444223-supplementary.pdf]

FITNESS TEST

Test session • 1st at16 weeks, • 2nd at 20 weeks, • 3rd at 24 weeks, • 4th at 28 weeks

Date ________________________

Name of dog ____

Date of birth/age ________________________

Weight ________________________

	BASE	POST1	POST2	REC	BL 5-8 min after 200 m sprint
HR					--------
BL				--------	
Time					
Running Speed	-------			--------	-------
Running time	-------			--------	-------

Notes:

Supplementary Figure 1. Form for use in recording measurement results.
